# The importance of clinical pharmacokinetic–pharmacodynamic studies in unraveling the determinants of early and late tuberculosis outcomes

**DOI:** 10.4155/ipk-2017-0004

**Published:** 2017-07-12

**Authors:** Andrew D McCallum, Derek J Sloan

**Affiliations:** 1 Liverpool School of Tropical Medicine, Pembroke Place, L3 5QA, Liverpool, UK; 2 Wellcome Trust Liverpool Glasgow Centre for Global Health Research, University of Liverpool, Liverpool L69 3GF, UK; 3 Malawi-Liverpool-Wellcome Trust Clinical Research Programme, Blantyre, Malawi; 4 School of Medicine, University of St Andrews, North Haugh, St Andrews, KY16 9TF, UK

**Keywords:** clinical trials, compartmental pharmacokinetics, multidrug-resistant tuberculosis, pharmacogenetics, pharmacokinetics–pharmacodynamics, therapeutic drug monitoring, tuberculosis

## Abstract

Tuberculosis remains a major infectious cause of morbidity and mortality worldwide. Current antibiotic regimens, constructed prior to the development of modern pharmacokinetic-pharmacodynamic (PK–PD) tools, are based on incomplete understanding of exposure–response relationships in drug susceptible and multidrug resistant tuberculosis. Preclinical and population PK data suggest that clinical PK–PD studies may enable therapeutic drug monitoring for some agents and revised dosing for others. Future clinical PK–PD challenges include: incorporation of PK methods to assay free concentrations for all active metabolites; selection of appropriate early outcome measures which reflect therapeutic response; elucidation of genetic contributors to interindividual PK variability; conduct of targeted studies on special populations (including children); and measurement of PK–PD parameters at the site of disease.

In 2015 there were an estimated 10.4 million new tuberculosis (TB) cases, 1.4 million TB deaths in HIV uninfected patients and 0.4 million TB deaths among people living with HIV [1]. Despite significant progress since the 1990s, the WHO End TB Strategy aim of ‘a world free of TB’ by 2035 will not be achieved without scientific advances to improve antituberculous chemotherapy. To evaluate the role of clinical pharmacokinetic (PK) and pharmacodynamic (PD) studies in reaching this target it is necessary to understand the problems with current treatment and review desirable attributes for new antibiotic regimens.

## Current problems & priority goals for TB therapeutics

Between 1946 and 1986, a series of multicenter clinical trials conducted by the British Medical Research Council and others demonstrated that patients suffering from drug-susceptible (DS)-TB, caused by *Mycobacterium tuberculosis* (Mtb) bacteria in the absence of antimicrobial resistance, could be cured by a 6-month course of combination therapy (2 months of rifampicin, isoniazid, pyrazinamide and ethambutol followed by 4 months of rifampicin and isoniazid) with a 5–8% post-treatment relapse rate at 2 years [2]. These data informed the design of current ‘short-course’ treatment regimens and TB seemed destined to become a disease of the past.

However, the late 20th century saw a dramatic TB resurgence with the major burden of illness in low and middle income countries. Particularly in sub-Saharan Africa, the co-existent HIV pandemic catalyzed a sharp rise in TB incidence, making adherence of every patient to 6 months of chemotherapy impossible for public health services to support [3]. To exacerbate matters, antituberculous drugs can cause significant toxicity and some components (principally rifampicin) are associated with problematic drug–drug interactions when combined with antiretroviral therapy [4]. Poor adherence and complex polypharmacy leads to unfavorable outcomes and it has become apparent that shorter TB treatment is needed [5]. Mathematical modeling suggests that shortening effective chemotherapy to 2 months could reduce TB incidence by 20%, and mortality by 25%. More modest gains could be achieved by treatment abbreviation to 4 months [6].

Linked to failures in DS-TB therapy, a marked increase has been observed in the incidence of multidrug resistant (MDR-) TB, caused by Mtb bacteria which are resistant to both rifampicin and isoniazid. The estimated number of MDR-TB cases worldwide rose from 250,000 in 2009 to 480,000 in 2015 [1]. Extensively drug-resistant TB, with super-added resistance to injectable second line drugs and fluoroquinolones, comprises 10% of MDR-TB cases. Some of the highest rates are in Eastern Europe where the collapse of health infrastructure during the disintegration of the Soviet Union contributed to difficulties for DS-TB treatment completion [7].

MDR-TB typically requires treatment prolongation to 18–20 months, although the WHO has approved the use of a 9- to 12-month regimen in some circumstances [8]. The second line drugs used for MDR-TB carry additional toxicities so the need for faster, cleaner therapy is even stronger than in drug susceptible disease [9,10].

The question for clinical pharmacologists is whether detailed interrogation of antibiotic exposure–response relationships will accelerate development of shorter, less toxic therapy for all forms of TB.

## A role for clinical PK–PD studies in achieving these goals

The clinical trial sequence that resulted in current short course chemotherapy for DS-TB was completed by 1985, and moved directly from Phase I studies of restricted scope to pivotal Phase III trials [2]. Modern PK–PD tools and population pharmacokinetic analysis had not yet been established [5]. Consequently, correlation between drug concentration and therapeutic effect was incompletely evaluated.

Subsequent preclinical experiments have derived summary PK measures, including the maximum drug concentration (C_max_) or area under the concentration-time curve (AUC), from murine [11,12] or hollow fibre system (HFS) models [13–18] of TB disease. These parameters may be expressed relative to the minimum inhibitory concentration (MIC) of each antibiotic for the infecting Mtb isolate, and related to the rate of bacterial clearance from the model. Results suggest that the efficacy of rifampicin, isoniazid, pyrazinamide and ethambutol is driven by AUC/MIC [13,15–17]. For rifampicin, isoniazid and ethambutol there is also a relationship between efficacy and C_Max_/MIC [15–17]. Data from retrospective meta-analyses contend that the PK–PD indices associated with efficacy in HFS studies are also relevant to clinical disease [18].

Alongside this thought-provoking preclinical PK–PD work sits a growing body of population PK literature revealing up to tenfold interindividual variability in plasma PK indices for first-line antituberculous drugs [19–22]. Studies comparing drug concentrations (particularly the C_Max_ of rifampicin) to a predefined reference range invariably showed antibiotic exposure to be unpredictable and lower than expected [23,24], giving rise to the argument that more detailed clinical PK–PD studies are needed to delineate the contribution of inadequate antibiotic exposure to unfavorable outcomes. Two potential benefits are cited for this approach; improved knowledge of the PK–PD drivers of treatment success may enable early phase clinical trials to predict whether dose escalation of key agents could facilitate treatment shortening in new standardized regimens, and greater understanding of early PK–PD targets linked to long-term cure may permit therapeutic drug monitoring (TDM) and intensification of therapy for patients at high risk of unfavorable outcomes.

## Clinical PK–PD study design

**Figure 1.**
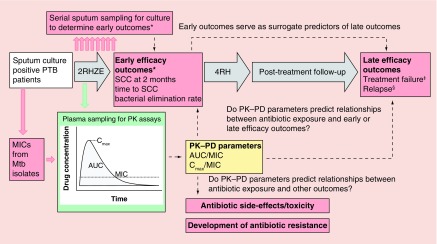
**Generic study design for clinical PK–PD study in DS-TB.** Procedures to generate PK parameters are shown in green. Procedures to generate PD parameters and study outcome measures are shown in red. PK–PD parameters which may be related to study outcome measures are shown in yellow. Number before drug combinations denotes intended duration of therapy in months. Other abbreviations are as described in the main text. *TB cultures to determine early efficacy measures may be set up in solid or liquid culture. ^‡^Treatment failure is normally defined as persistently positive sputum cultures until the end of TB therapy. ^§^Relapse is normally defined as cure (negative sputum culture) at the end of TB therapy, but reversion to positive cultures with the same Mtb strain as the baseline isolate during post-treatment follow-up. E: Ethambutol; H: Isoniazid; MIC: Minimum inhibitory concentration; Mtb: *Mycobacterium tuberculosis*; PTB: Pulmonary tuberculosis; R: Rifampicin; SCC: Sputum culture conversion; Z: Pyrazinamide.

A generic design to provide an overview of clinical PK–PD studies in TB is outlined in Figure 1. For illustration, only the current 6-month treatment regimen for DS-TB is shown but timescales and regimens may be adapted for MDR-TB. PK–PD studies nested within early phase clinical trials may compare multiple drugs and dose combinations.

Blood sampling for plasma or serum PK assays is generally drawn after daily medications have reached a steady state concentration cycle, typically after at least 14 days of therapy. Intensive PK sampling to establish the AUC for the 24 h after dosing (AUC_0–24_) is costly and labor intensive because a minimum of six or seven blood samples must be collected at carefully recorded time-points. All specimens should be promptly centrifuged so that plasma or serum can be harvested and frozen at 80°C, prior to batched analysis by HPLC and mass spectrometry. For studies in TB endemic, resource poor countries bioanalysis often requires cold shipment to distant laboratories. Novel approaches to measuring drug exposure or activity in resource-limited settings include the use of dried blood spot methods to store and transport samples [25–27], urine colorimetry to detect low rifampicin exposure [28] or co-culturing patients’ Mtb isolate with their plasma on treatment in liquid culture to give an indication of the relative activity of the treatment regimen [29]. For some antituberculous drugs (e.g., amikacin [30], kanamycin [30], moxifloxacin [31] and linezolid [32] in MDR-TB) limited plasma sampling strategies have sought to determine which single time-point measurements best represent more complex PK indices. For other antibiotics (e.g., rifampicin) Bayesian statistical techniques have been deployed to calculate AUCs from sparse sampling strategies (often two to three blood draws) [33]. Potential drawbacks to the Bayesian approach are the need for prior intensive PK data from the same population and reliance on sophisticated computer software [34]. There is also a lack of consensus on optimum sparse sampling time-points for some drugs.

As with preclinical studies, it is recognized that antibiotic PK parameters such as AUC and C_max_ should be related to the MIC of each drug for the infecting Mtb isolate. In fact, lack of detailed epidemiological data on MIC distributions for Mtb from most high-incidence TB settings [35,36] makes calculation of AUC/MIC and C_Max_/MIC more important for clinical pharmacology studies than for murine or HFS models. Mtb MIC assay plates covering most first and second line antituberculous drugs are available which may expand access to PK–PD testing for TB patients [37], and there have been some practical applications of this approach [38,39]. Correlation of specific genetic mutations in the Mtb genotype with MIC range for some drugs (e.g., *gyrA* mutations and fluoroquinolone MICs) may also provide more rapid, detailed information than is currently available on the complexity of variable drug susceptibility in the clinical setting [40].

Once PK–PD parameters have been established, selection of appropriate outcome markers is not straightforward. While some studies focus on toxicity or induction of antibiotic resistance, a common purpose is to identify relationships with treatment efficacy. Strengths and weaknesses of TB efficacy end points in regular use by clinical researchers are outlined in Table 1.

**Table 1.** **Treatment efficacy outcomes used in clinical pharmacokinetic-pharmacodynamic studies and clinical trials for tuberculosis.**

**Timing of outcome measurement**	**Outcome measurement**	**Strengths**	**Weaknesses**
Late (typically the end of post-treatment follow-up)	Treatment failure or post-treatment relapse	1. Clinically relevant (target PK–PD parameters for TDM would ideally be validated against this outcome) 2. Gold standard end point for stable cure in Phase III clinical trials	1. Studies are very long and expensive to conduct 2. Unless careful molecular tests can be undertaken the end point of relapse may be contaminated by TB re-infection in highly endemic settings, especially with high rates of HIV co-infection

Early (typically 2 months for DS-TB, may be 24 weeks for MDR-TB)	SCC^†^ at a defined end point	1. Results are simple to understand and interpret 2. Only requires sputum sampling at two time-points	1. Only modest correlation with late outcomes 2. Binary data do not use all information (e.g., 2-month results cannot discriminate between patients who are culture negative at 8 weeks, even if one culture converted at 2 weeks and the other at 6 weeks)
	Time to SCC^†^	1. Results are simple to understand and interpret 2. Provides more discriminatory data than SCC	1. Correlation with late outcomes has not been well validated 2. Weekly sputum sampling required; wider sampling windows reduce the accuracy of the measurement
	Statistical modeling of bacterial elimination rates from serial quantitative bacteriology data^‡^	1. Provides information on antimicrobial efficacy across the whole sampling time, even on patients who do not convert to negative 2. Allows multiphase patterns of bacterial clearance to be assessed	1. Correlation of summary parameters from mixed effects modeling with late outcomes has not been well validated 2. Data analysis is computationally complex to perform 3. Results are not always simple to understand and interpret

^†^SCC or ‘time to SCC’ data can be generated using solid or liquid culture media; culture conversion is often later in liquid culture systems.

^‡^Quantitative bacteriology data can be generated from log_10_CFU/ml counts on solid media to Time to Positive results in liquid culture systems.

DS-TB: Drug-susceptible TB; MDR-TB: Multidrug resistant TB; PK–PD: Pharmacokinetic–pharmacodynamic; SCC: Sputum culture conversion; TDM: Therapeutic drug monitoring.

The risk of TB relapse after completion of therapy is well recognized, so definitive Phase III clinical trials in DS-TB conventionally use a late efficacy outcome of relapse-free cure 18–24 months after enrollment as their gold standard end point [2,41]. TDM studies seeking to identify and validate PK–PD targets linked to long-term cure would also ideally be validated against this late outcome. However, trials of this duration, or longer for MDR-TB, are complicated and expensive to complete [10]. From this perspective, clinical PK–PD analyses based on earlier efficacy outcomes in Phase II trials may help to compare novel combinations of drugs and dosages using fewer patients over a shorter timeframe so that only candidate regimens with the greatest efficacy and treatment shortening potential advance to Phase III evaluation.

For this approach, the early outcomes of Phase II studies should predict relapse. Traditionally, for DS-TB patients with pulmonary TB, sputum culture conversion (SCC) from ‘positive’ to ‘negative’ at two months is used to represent treatment efficacy [42]. However, this simple biomarker shows only modest correlation with late outcomes [41,42]. In recent years, attention has shifted toward weekly sputum sampling to more accurately record the ‘time to culture conversion’ and provide more information on the antibacterial effect of therapy over the entire study period than binary measurement at a single time-point [43,44]. Modern studies have also seen a gradual move from microbiological treatment monitoring on solid (e.g., Lowenstein-Jensen [LJ]) media to liquid culture (e.g., the mycobacterial growth indicator tube system) because liquid Mtb culture is easier, provides faster results and converts to ‘negative’ later during therapy [45,46]. It is generally believed that TB relapse is driven by drug-tolerant bacteria which survive despite antibiotic therapy [5]. Therefore, while the PD of distinct bacterial phenotypes in different media are incompletely understood [47], it seems intuitive that liquid culture systems which revive ‘persister’ organisms for longer will provide better surrogates of final outcomes [48,49]. Some reports indicate that liquid culture may still fail to revive a proportion of differentially culturable ‘persister’ cells, which only grow from expectorated sputum when media is augmented with exogenous culture filtrate (CF) or resuscitation-promoting factors [50,51]. The effect of antibiotic therapy on CF- or resuscitation-promoting factors-dependent bacilli may also be important determinants of TB treatment response, but the relationship between survival of these cells and clinical outcomes has not been evaluated.

Newer methods to monitor TB treatment response and set early outcome measures for PK–PD studies involve use of serial quantitative bacteriology to chart the decline in Mtb bacillary load. In solid media, incubation of homogenized sputum onto on selective agar plates (e.g., Middlebrook 7H10 or 7H11) allows the number of colony forming units (usually expressed in log_10_CFU/ml) in each expectorated patient sample to be counted [52–54]. For liquid culture, the time to positivity after inoculation of processed sputum into Mycobacterial Growth Indicator Tubes provides an inverse measure of bacillary load (usually expressed in hours or days) because shorter delay until detection of growth represents higher bacterial burden [53,55–56]. Early bactericidal activity studies lasting up to 14 days directly report changes from baseline in log_10_CFU/ml counts or time to positivity [48,57], while studies extending out for 8–12 weeks may deploy statistical modeling techniques to summarize evolving patterns of bacillary clearance over a longer period [53,58–59]. Separate elimination rates can be reported for distinct ‘early bactericidal’ and ‘sterilizing’ phases of treatment [43,60] and correlation has been described between sterilization phase rate co-efficients and long-term DS-TB outcomes [53]. More data are required to validate those relationships.

Presently, no early TB outcome measure convincingly predicts long-term relapse and there is no consensus on which to use. This is demonstrated by the recent failure of three multicenter Phase III trials to shorten drug-susceptible TB treatment from 6 to 4 months by inclusion of a fluoroquinolone [61–63] despite promising results from Phase II clinical studies which used end-points of 2-month SCC [45,64–65], time to culture conversion [65] and bacillary elimination rate [59]. Therefore, PK–PD studies relating antibiotic exposure to early outcomes must be aware of the potential limitations to their findings. Future clinical PK–PD analyses may be crucial to development of better treatment response biomarkers.

## Existing clinical PK–PD data

Table 2 describes published studies which have adopted a PK–PD approach to relate plasma or serum antibiotic exposure to DS-TB treatment efficacy in adults. The collective results are difficult to interpret; some studies report an association between PK variability and treatment response [66–74] while others do not [75–79]. No PK–PD markers consistently emerge as potential predictors of efficacy in Phase II clinical trials, or targets to inform TDM strategies in clinical practice.

**Table 2.** **Summary of pharmacokinetic–pharmacodynamic studies to evaluate antibiotic exposure-treatment efficacy relationships in adults with drug-susceptible tuberculosis.**

**Study (year), site**	**n =**	**PK sampling**	**PK parameters**	**MICs**	**Outcome measures**	**Results**	**Ref.**
Narita (2001), USA	69	RH 2, 6 h	C_Max_	No	**Late:** TB recurrence (presumed relapse) after completion of therapy	No effect of R or H PK on t outcome	[76]

Weiner (2003), USA	133	Rp, H 1, 2, 5, 24 h	C_Max_ AUC_0-12 h_	No	**Late:** Composite outcome of treatment failure and relapse	Lower Rp and H AUC_0-12h_ associated with poor outcome	[67]

Weiner (2003), USA	102	Rp, H 1, 2, 3, 6, 24h	C_Max_ AUC_0-12h_ AUC_0-24h_	No	**Late:** Composite outcome of treatment failure and relapse Development of rifamycin resistance	Lower Rb AUC_0-24h_ associated with poor outcome and development of antimicrobial resistance Lower H AUC_0-12h_ associated with poor outcome and development of rifamycin resistance	[66]

Ribera (2007), Spain	22	RH 0.5, 1.5, 2, 3, 4, 6, 8, 12h	C_Max_ AUC_0-24h_	No	**Late:** Composite outcome of treatment failure and relapse	No effect of R or H PK on outcome	[79]

Chang (2008), Hong Kong	72	R only 2, 4h	C_max_	No	**Early:** 2-month SCC	No effect of R PK on 2-month SCC	[75]

Chideya (2009), Botswana	225	RHZE 1, 2, 6 h	C_Max_ AUC_0-6 h_	No	**Late:** Composite outcome of treatment failure or death during therapy	Low C_Max_ for Z (<35 mg/l) associated with poor outcome	[68]

Burhan (2013), Indonesia	167	RHZE 2hr^†^	C_2 h_	No	**Early:** 2-month SCC Additional post-hoc of ≥ 1 positive sputum culture at 4, 8 or 24 weeks	No effect of R, H or E PK on 2-month SCC Low C_2h_ for Z (<35 mg/l) associated with ≥ 1 positive sputum culture at 4, 8 or 24 weeks	[69]

Pasipanodya (2013), South Africa	142	RHZ 0.5, 1, 1.5, 2, 2.5, 3, 4, 6, 8h	C_Max_ AUC_0-24h_	No	**Early:** 2-month SCC **Late:** Composite outcome of treatment failure, death and relapse	Low C_Max_ for Z (<58.3 mg/l) most strongly associated with reduced 2-month SCC Low AUC_0-24h_ for R (<13 mg.h/l), H (<52 mg.h/l) and Z (<363 mg.h/l) associated with poor long-term outcomes	[70]

Chigutsa (2014), South Africa	154	RHZE 4-8 samples over 7 h	C_Max_ AUC_0-24h_	Yes	**Early:** 8-week sputum bacterial elimination rates^‡^	Low C_Max_ for R (<8.2 mg/l) and low AUC_0-24h_/MIC (<11.3 mg.h/l) associated with slower bacterial elimination	[71]

Prahl (2014), Denmark	32	RHZE 2h	C_2h_	No	**Late:** Treatment failure	Treatment failure more common with low C_2h_ of both R (<8 mg/l) and H (<3 mg/l)	[72]

Requena-Méndez (2014), Peru	113	H 2, 6h	C_2h_	No	**Late:** Composite outcome of treatment failure and relapse	No effect of H PK on outcome	[78]

Sloan (2014), Malawi	133	RHZE 2, 6h	C_Max_ AUC_0-6h_	No	**Early:** 2-month SCC **Early:** 8-week sputum bacterial elimination rates^§^ **Late:** Composite outcome of treatment failure and relapse	Lower AUC_0-6h_ for H and Z associated with reduced 2-month SCC Low AUC_0-6h_ for H (<15 mg.h/l) associated with slower bacillary elimination Lower AUC_0-6h_ for H associated with poor long term outcomes	[73]

Mah (2015), Canada	134	RH 1-2, 6h	C_Max_	No	**Early:** 2-month SCC	Low C_Max_ of H (<3 mg/l) associated with reduced 2-month SCC	[74]

Park (2015), South Korea	413	RHZE 2h	C_2h_	No	**Early:** 2-month SCC **Late:** Composite outcome of treatment failure and relapse	No effect of R, H or E PK on 2-month SCC or late outcome	[77]

^†^Full PK profile done on a subset of nine patients.

^‡^Bacterial elimination rates based on time to event modeling from liquid culture data [80].

^§^Bacterial elimination rates based on mixed effects modeling from solid and liquid culture data [53].

E: Ethambutol; H: Isoniazid; MIC: Minimum inhibitory concentration; R: Rifampicin; Rb: Rifabutin; Rp: Rifapentine; SCC: Sputum culture conversion; Z: Pyrazinamide. Other abbreviations are as described in the main text.

There are several potential explanations for these mixed results. Pharmacological variability is one of many factors influencing TB treatment [34] and may have varying impact in different settings. The published studies suffer from considerable heterogeneity in design and execution. Most reported antibiotic concentrations at one or two time-points only, reducing the accuracy of C_Max_ and AUC calculations. Only one related PK indices to MICs from infecting Mtb isolates [71]. A wide range of early and late treatment outcomes measures were used. Treatment failure and relapse are relatively rare events, so smaller cohorts may have been insufficiently powered to demonstrate the effect of antibiotic exposure on unfavorable outcome rates. Finally, the majority were single-arm observational studies which may have diminished their ability to detect relationships with treatment efficacy; for example, most of the interindividual variability in rifampicin concentrations in African settings was at the lower end of the likely exposure–response curve [23–24,73], making it difficult to demonstrate the incremental benefit of higher concentrations on steeper sections of the curve. Standardization of clinical PK–PD study design may provide clarification and facilitate meta-analysis of data from different sites [81].

So far this review has argued that modern PK–PD methods may improve TB treatment but more work is required to translate PK–PD data into practically useful information for researchers and clinicians. The next sections will provide specific examples of ongoing work where PK–PD studies are proving beneficial.

## Ongoing PK–PD work in DS-TB

DS-TB compromises 95% of TB cases worldwide, with a public health approach required to manage large numbers of patients in low resource settings. Therefore, the main utility of PK–PD work in DS-TB will likely be for development of new, dose-optimized, standard regimens rather than individual patient TDM.

The clearest example of an antibiotic for which dose and exposure–response relationships require re-analysis is rifampicin, a key sterilizing mycobacterial RNA synthesis inhibitor which is critical for achieving relapse-free cure with current DS-TB regimens [2].

The current rifampicin dose (10 mg/kg once daily) was selected in the 1960s to facilitate 6-month treatment while minimizing expense [82]. Even when costs dropped, toxicity concerns discouraged dose escalation. However, preclinical experiments now suggest that increased rifampicin doses (up to 160 mg/kg/day) may be tolerable and could shorten treatment [16,83]. Monotherapy studies in humans have demonstrated a steeper fall in bacterial load over 2–14 days with modest dose increases [48,84], and a systematic review of trials including rifampicin doses up to 20 mg/kg showed an association between higher doses and faster SCC [82]. In 2015, a maximum tolerated dose study from Cape Town reported rifampicin dosing up to 35 mg/kg without any limiting toxicity so further work, beginning at 50 mg/kg is planned [85]. Data from Bolivia, Nepal and Uganda corroborate the absence of rifampicin toxicity to 20 mg/kg [86]. The accumulative evidence illustrates a need for new clinical PK–PD studies of high dose rifampicin use.

Intensive PK sampling and analysis from the Cape Town study showed that increased rifampicin doses caused ‘super-proportional’ increases in plasma antibiotic exposure; doubling the dose from 10 to 20 mg/kg was associated with a more than fourfold increase in AUC_0–24h_, the average AUC_0–24h_ at 35 mg/kg was almost 10-fold higher than with standard dosing and the lowest recorded AUC_0–24h_ and C_Max_ increased with almost every dose step [85]. Although not powered to detect differences in microbiological efficacy, there was a trend toward faster day 14 sputum bacterial clearance rates on solid and liquid media at 35 mg/kg than at lower doses and the AUC_0–24h_ of rifampicin at day 14 was a better predictor of 14-day bactericidal activity than the dose administered (either in ‘mg’ or weight-adjusted as ‘mg/kg’) [85].

A further Multi Arm Multi Stage randomized Phase II trial of several novel treatments for DS-TB (including high-dose rifampicin, moxifloxacin and the experimental compound SQ109) in South Africa and Tanzania has since shown reduced time to sputum culture conversion on liquid media at rifampicin 35 mg/kg across a period of 12 weeks. A 20-patient PK sub-study from that cohort confirmed that rifampicin dose escalation has a ‘super-proportional’ plasma drug exposure effect [87]. Additional PK–PD insights may be gleaned from that dataset, and two more Phase II trials on the efficacy of high dose rifampicin are yet to report (www.clinicaltrials.gov NCT01408914 in Peru and NCT00760149 in Tanzania).

While these data are encouraging, caution is required. Most high dose rifampicin studies excluded HIV co-infected patients or selectively recruited antiretroviral therapy naive individuals with high CD4 counts. Severely immunocompromised HIV patients may be at higher risk of poor drug absorption or adverse events and, as rifampicin is a potent inducer of cytochrome P450 enzymes, higher doses may present an increased challenge in the management of drug–drug interactions. Future clinical PK–PD studies should investigate exposure–response on TB outcomes in this vulnerable population [4]. Furthermore, the notorious unreliability of early PD markers at predicting relapse precludes confidence that rifampicin dose escalation will permit treatment shortening until completion of definitive trials. One Phase III study, (RIFASHORT, NCT02581527) will start recruiting soon with experimental regimens containing 1200 and 1800 mg of rifampicin (20–30 mg/kg for 60 kg adults). While these data will be valuable it remains to be seen whether this level of dose escalation is enough, particularly as current PK–PD and toxicity data suggest scope to go higher.

PK–PD and dose escalation studies have been undertaken on other rifamycins. Rifapentine produces higher serum concentrations than rifampicin after 10 mg/kg oral dosing and has a longer half-life (15 h, compared with 2–3 h for rifampicin) [34]. Dose increases to 20 mg/kg have been tolerated by healthy volunteers, although the rises in plasma AUC_0–24h_ associated with higher doses were not super-proportional [88]. Clinical trials including rifapentine are ongoing (TBTC Study 31, NCT02410772). Rifabutin is sometimes substituted for rifampicin in patients with high drug–drug interaction risks because it is less potent inducer of cytochrome P450 enzymes [89]. Its absorption is variable in HIV patients [90] and toxicities (including leucopenia and uveitis) are concentration dependent. TDM is highly recommended and dose increases may be problematic.

Alongside the rifamycins, administration of pyrazinamide for the first 2 months of treatment is key to the sterilizing efficacy of 6-month first-line regimens for DS-TB [2]. Pyrazinamide primarily exerts antituberculous activity in acidic conditions. While early bactericidal activity studies with pyrazinamide demonstrated poor independent bactericidal activity over 14 days, it enhanced the activity of other drugs [48] and some clinical PK–PD studies have shown that reduced exposure is associated with poor late outcomes [68–70]. The population PK profile of pyrazinamide is more stable than rifampicin or isoniazid so predictable plasma concentrations are usually achieved [20,24]. The currently recommended dose is 20–30 mg/kg. Concerns about side-effects of arthralgia and dangerous hepatotoxicity have stifled calls for dose escalation. However, preclinical data suggest that doses up to 60 mg/kg could improve efficacy [13]. A meta-analysis of dose-toxicity relationships in clinical studies reported that, although arthralgia is dose-related, higher dose pyrazinamide did not significantly increase hepatotoxicity and some adverse liver events may be idiosyncratic [91]. Computer simulations suggest that modest pyrazinamide dose increases (up to 40 mg/kg) alongside higher dose rifampicin may accelerate 2-month SCC. Clinical trials with nested PK–PD analyses and toxicity monitoring are planned to test this proposition.

Pharmacological lessons on optimal dosing and drug–drug interactions may yet emerge from the Phase III fluoroquinolone trials which failed to shorten DS-TB therapy [61–63]. A PK sub-study of the OFLOTUB trial [61] identified that gatifloxacin exposure decreased with rifampicin, isoniazid and pyrazinamide co-administration; and that double-dose gatifloxacin may optimize the bactericidal effect and reduce the probability of resistance [92]. Similarly, drug–drug interactions between rifampicin and isoniazid and moxifloxacin reduce moxifloxacin C_max_ and AUC by 32 and 31%, respectively [93]. Refinement of fluoroquinolone dosing through PK–PD studies may improve outcomes in future trials.

## Ongoing PK–PD work in MDR-TB

**Figure 2.**
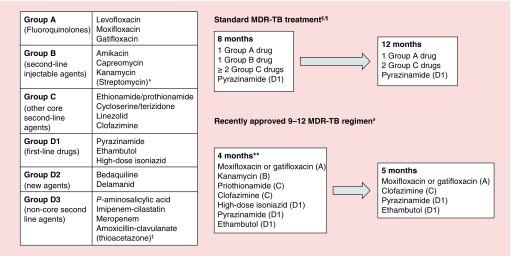
**WHO-approved MDR-TB treatment regimens. MDR-TB regimens combine second-line drugs from the panel on the left.** *Streptomycin may substitute for other injectable agents in specific scenarios. ^‡^Thioacetazone must not be used in HIV co-infection. ^§^This template should be adapted to the antimicrobial resistance profile of the infecting Mtb isolate. If a regimen containing 5 drugs which are likely to be effective cannot be constructed additional group D1-3 agents should be added. ^¶^Most patients will require a second-line injectable agent for 8 months and total treatment for 20 months but there is scope for modification according to patient response. ^#^The 9-12 regimen may only be used when resistance to fluoroquinolones and second-line injectable agents has been excluded or is considered unlikely. **Patients who do not adequately respond to therapy (e.g., sputum smear conversion by 4 months) may have the intensive phase of therapy extended.

Treatment of MDR-TB is more difficult than DS-TB, with lower treatment success rates and higher incidence of treatment limiting toxicity [9]. Traditional and emerging regimens combine new compounds with old second-line drugs [8] (see Figure 2) and the evidence to support optimal dose combinations is sparse. There is, therefore, considerable scope for clinical PK–PD studies to support optimization of standard drug dosing and TDM. Due to space constraints, two illustrative examples are provided here.

The oxazolidinone, linezolid, has recently been re-classified as a ‘core second line agent’ for MDR-TB treatment [8] (see Figure 2) and may be particularly important for patients with extensive antimicrobial resistance [94–96]. Prolonged therapy is complicated by serious side-effects; bone marrow suppression and peripheral or optic neuropathy are reported in 28% of patients [8]. The routine dose (600 mg orally, twice daily), licensed for treatment of Gram positive nonmycobacterial infections, is currently being formally evaluated alongside bedaquiline and pretomanid in the Nix-TB trial (NCT02333799). To increase tolerability doses of 600 mg daily, 300 mg twice daily and 300 mg once daily are often administered under programmatic conditions [97] and a 600 mg daily dose will be assessed as part of several five drug regimens in the NEXT-TB trial (NCT02454205). A systematic review has tentatively indicated that 300 mg twice daily daily may retain efficacy while avoiding toxicity but there were difficulties in selecting end points for this retrospective PK–PD analyses [98]. Prospectively collected data are required and recruitment is ongoing to a dose ranging study in South Africa.

Although it is hoped that all-oral treatment for MDR-TB will be available in the future, aminoglycosides (amikacin and kanamycin) are currently integral to all approved regimens. Longer duration, higher doses of these drugs (WHO recommended range: 15–20 mg/kg/day up to a maximum of 1000 mg daily [8]) contributes to favorable outcomes [99] but the therapeutic index is narrow; nephrotoxicity and irreversible ototoxicity are common [100]. TDM is traditionally recommended to guide aminoglycoside therapy by assaying ‘peak’ and ‘trough’ drug concentrations. However, recent HFS [101] experiments and clinical PK–PD studies in Botswana [102] have suggested C_Max_/MIC and AUC_0–24h_/MIC to be better predictors of treatment efficacy (the clinical studies were based on sputum culture conversion) while cumulative AUC_0–24h_/MIC and duration of therapy correlate best with the risk of ototoxicity [103]. In the Netherlands, individualized aminoglycoside dosing based on C_Max_/MIC resulted in patients receiving an average dose of 6.7 mg/kg [30], which is significantly lower than the WHO recommendation. A retrospective evaluation of the regimen incorporating this approach showed favorable results [104]. Further studies are required but this saliently illustrates how suitable PK–PD thresholds might support safer individualized therapy based on TDM.

Clinical pharmacologists may also help to address further MDR-TB therapeutics issues, including: the role of higher fluoroquinolone doses for patients with resistance mutations conferring modestly elevated MICs [40,105]; the synergistic or antagonistic effects of new antituberculous compounds in combination; and exposure-toxicity aspects of electrocardiographic QTc prolongation by drugs such as fluoroquinolones, clofazimine, bedaquiline and delamanid [106]. There is insufficient space for detailed discussion of these. The remainder of this review will consider emerging challenges and opportunities for the design and conduct of future PK–PD studies.

## New assays & outcome measures

Practical aspects of PK sample collection and processing have already been outlined. An additional consideration is that several antibiotics are metabolized to other active compounds after administration (e.g., rifampicin is converted to 25-desacetylrifampicin and pyrazinamide is the pro-drug of pyrazinoic acid [107]). Assays of all active metabolites may be needed to understand the PK–PD characteristics of such agents.

Limitations of sputum culture-based early TB outcome measures have also been discussed. A molecular bacterial load assay has recently been developed which reports the Mtb bacterial load by quantitation of 16S ribosomal RNA within 72 h of sample processing [108]. This is much faster than the turnaround time for culture. Changes in the molecular bacterial load assay on therapy may allow more efficient TB treatment monitoring [109] and help to develop better outcome measures for TDM and clinical trials. The impact of this technique will depend on validation of data against long-term relapse.

## Interindividual variability & special populations

Wide interindividual variability in the plasma exposure of some antituberculous antibiotics partly underpins the rationale for clinical PK–PD studies [19–22]. Co-morbidities, concomitant medications, dietary intake and genetic factors regulating drug metabolism all contribute. Greater understanding of the role played by genotype may identify patients at high risk of low exposure, potentially focusing TDM and earlier intervention to improve outcomes.

Isoniazid clearance is driven by *N*-acetyltransferase 2 enzymes in the liver and small intestine, regulated by the polymorphic *NAT2* gene [110]. Single nucleotide polymorphisms in several *NAT2* alleles confer fast or slow acetylation phenotypes [110,111]. Low isoniazid exposure in fast acetylators may be associated with increased risk of unfavorable outcomes and acquired drug resistance while high exposure in slow acetylators may be associated with toxicity [111]. Given the dominant effect of *NAT2* genotype on isoniazid exposure, practicing clinicians and researchers should consider categorizing patients by acetylator status.

Genotypic drivers of rifampicin variability are less clear. Biliary excretion occurs after hepatocellular uptake, primarily mediated by organic anion-transporting polypeptide 1B1 coded for by the gene *SLCO1B1* [112]. Recent reports from South Africa and Uganda suggest that single nucleotide polymorphisms in *SLCO1B1* are more common in African patients and associated with up to 28% reduction in rifampicin AUC_0–24hr_ in homozygotes [112,113]. However, given extensive population diversity in *SLCO1B1* polymorphism carriage [114], these findings have not been replicated in India, Tanzania or Malawi [115,116,117]. Future PK–PD studies should investigate further.

Some PK variability is associated with physiologically distinct, but previously neglected populations where a strong case exists for separate PK–PD studies and clinical trials. Foremost among these are children. Previously, pediatric dosing and duration of first-line TB therapy were extrapolated on a mg/kg basis from adult studies, but this resulted in lower plasma concentrations than the adult population. In 2010, the WHO recommended increased doses of all four first-line TB drugs [118]. Few PK studies have been performed to assess the impact of these adjustments. Emerging data from South Africa and Malawi indicate that rifampicin exposures remain low [119,120]. A cohort evaluation of 161 Indian children aged 1–15 has suggested that a low C_Max_ for rifampicin (<3.01 mg/l) or pyrazinamide (<38.01 mg/l) predicted late outcomes of treatment failure or death [121]. There is almost no clinical PK–PD information on second-line drugs for MDR-TB in pediatric populations.

Although the evidence is debatable, patients with HIV co-infection [23,122] diabetes mellitus [123–125] and pregnant women [126] may also have unusual PK–PD profiles, necessitating detailed sub-studies to clarify the role of TDM and altered antibiotic dosing. Systematic use of early TDM with dose correction in diabetic patients may shorten time to sputum culture conversion [127].

## PK–PD at the site of disease

All the PK–PD data described hitherto report antibiotic exposure in peripheral blood, but TB is not primarily a bloodstream infection. In pulmonary TB, Mtb exists within discrete microenvironments: intracellular organisms within granuloma macrophages; free extracellular organisms in caseum, cavities and airways; and in apparently ‘normal’ interstitial lung tissue [128]. It is improbable that unbound drug passively equilibrates between blood and all tissue compartments so investigation of relationships between peripheral blood and lesion PK–PD is important. Advantages and disadvantages of clinically focused methods for this are outlined in Table 3.

**Table 3.** **Pharmacokinetic sampling sites.**

**Sampling site**	**Advantages**	**Disadvantages**
Peripheral blood (plasma or serum)	Ease of repeat sampling Multiple/rich PK sampling possible Most existing data for comparison are from these samples	Far from site of infection Unclear relationship with outcome Wide interindividual variability

Bronchoalveolar lavage	‘Near infection’ samples - alveolar macrophages and epithelial lining fluid Can be paired with rich plasma/serum sampling Models of intrapulmonary exposure can be developed using population modeling techniques	Invasive procedure Single time point sampling Sample from alveoli and bronchioles rather than within granuloma Drug loss due to efflux when sampling Differential penetration depending on inflamed/noninflamed lung/different lobes Sampling retrieves mixture of macrophages, T-lymphocytes and epithelial cells

Lung explant studies	Can assess spatial drug penetration Can combine with spatial data on drug susceptibility profile and MIC	Only possible in patients requiring lung resection (either severe disease or a sub-set of MDR-TB patients) Single time point drug concentrations

Cerebrospinal fluid (for TB meningitis)	Accessible Can be paired with rich plasma sampling Models of CSF drug exposure can be developed using population modeling techniques	Invasive procedure Single time point sampling Traumatic tap will contaminate samples

CSF: Cerebrospinal fluid; MDR: Multidrug resistant; MIC: Minimum inhibitory concentration.

One novel approach is to generate spatial drug penetration information by performing matrix-assisted laser desorption/ionization mass spectrometry on dissected tissue from patients undergoing lung resection surgery for antibiotic-refractory disease [5,129]. Early results suggest excellent rifampicin and pyrazinamide penetration into the central necrotic caseum of TB granulomas but peripheral intracellular accumulation of moxifloxacin [130]. These data may explain why rifampicin and pyrazinamide are critical DS-TB sterilizing agents but treatment shortening trials with moxifloxacin were unsuccessful [62,63]. However, an alternative approach using microdialysis on *ex vivo* pulmonary cavities from patients with MDR-TB shows excellent penetration of levofloxacin into cavity walls, and good correlation between serum and cavitary fluoroquinolone concentrations [131]. From a PD perspective, lung resection studies have shown differing drug susceptibility patterns in Mtb isolates from different pulmonary cavities of the same patient [132,133] and mathematical models suggest that bacilli in compartments with low antibiotic penetration develop higher MICs. Overall, such advanced PK–PD work on resected lung tissue is beginning to generate very detailed information on the extent and consequences of antibiotic exposure at the site of infection. A disadvantage of these experiments is that they are only possible on a small sub-set of unusual and highly selected patients with very severe TB and particularly distorted lung anatomy.

An alternative means of ‘near infection’ PK sampling is the use of broncho-alveolar (BAL) lavage to sample epithelial lining fluid (ELF) and alveolar macrophages. Given the invasive nature of this procedure, all previous BAL studies were conducted on small numbers of healthy volunteers with single time-point sampling. Results show extensive variability in the pulmonary penetration of first-line drugs. In ELF, isoniazid concentrations appeared low in relation to likely MICs for clinical Mtb isolates and projected rifampicin AUC/MIC measurements appeared insufficient to suppress resistance in a high proportion of subjects [134–138]. Alveolar macrophage/ELF concentration ratios ranged from 0.1 to >20, with rifampicin, ethambutol and fluoroquinolones concentrating most effectively within cells [5,136,139]. Difficulties for BAL-based studies are the potential clinical hazard and infection control risks associated with performing bronchoscopies on sick patients and the need to collect luminal specimens rather than those directly from granulomas. An ongoing clinical PK–PD cohort of TB patients with BAL sampling in Malawi will provide new information.

Fifteen to twenty percent of TB cases are extra-pulmonary. Penetration of antibiotics to other infection sites may differ from the lungs. Specific PK–PD studies have been completed in TB meningitis, where treatment regimens derived from data on pulmonary TB do not account for the extent of drug penetration across the blood–brain barrier. Isoniazid, pyrazinamide and the fluoroquinolones reach cerebrospinal fluid (CSF) in high concentrations, whereas streptomycin does not [140]. Interestingly, for fluoroquinolones both ‘low’ and ‘high’ CSF drug exposures are associated with poorer outcomes, and intermediate exposures are associated with less death and disability [141]. It is likely that more extensive breakdown of the blood–brain barrier with severe disease explains the link between higher drug exposure and poorer outcomes.

CSF rifampicin exposure is lower than plasma [142]. A Phase II clinical trial of rifampicin (13 mg/kg) intravenously alongside moxifloxacin, isoniazid, pyrazinamide and corticosteroids in Indonesia identified a survival benefit from higher CSF rifampicin concentrations, with substantially lower 6-month mortality in patients receiving the higher dose [143,144]. Conversely, a Phase II trial in Vietnam, using oral rifampicin (15 mg/kg) and levofloxacin (20 mg/kg) did not improve survival [145]. More studies are needed to determine whether rifampicin dose escalation, perhaps allied to intravenous administration, does improve outcomes [85,146].

## Conclusion

Current treatment regimens for DS-TB and MDR-TB are not based on a detailed understanding of exposure–response relationships for the antibiotic combinations which are used. Development of shorter, less toxic therapeutic strategies for all forms of TB is a key global research priority. Preclinical studies in animals and HFS have indicated that clinical PK–PD studies may contribute toward achievement of this goal by helping to optimize dosing for new standard regimens and, in some cases, by providing an evidence base for TDM to guide the management of individual patients.

## Future perspective

After many barren years in TB drug development, intensified efforts to accelerate the evaluation combinations of existing antibiotics alongside novel compounds are beginning to bear fruit and there are real opportunities to improve treatment outcomes. PK–PD science will make an important contribution to this endeavor, increasing the chance of success, especially if insights from preclinical HFS work can be validated and extended by clinical PK–PD studies.

The greatest impact may be obtained at the level of Phase II clinical trials. If analysis of PK–PD parameters in relation to early treatment outcomes (measured ∼8 weeks into treatment for DS-TB) could predict the long-term risk of eventual relapse, more efficacious regimens might advance more quickly to Phase III evaluation. This would accelerate progress toward shorter therapy with attendant benefits for all patients. However, early treatment outcomes are currently poor surrogates of final end points and it remains to be seen whether new monitoring tools will help overcome this obstacle. Standardization of PK–PD study design and meta-analyses of larger total datasets may pave the way forward [81].

TDM may be of value in patients from groups with unpredictable PK characteristics (e.g., in the presence of HIV infection, potential drug–drug interactions or renal/hepatic dysfunction). It may also improve the treatment of MDR-TB where several drugs have a narrow therapeutic index. For this approach to be effectively exploited, plasma concentration ranges associated with safe and successful TB treatment need to be better defined. That is currently problematic, given the scarcity of PK–PD datasets for second-line antituberculous drugs but this knowledge gap provides fertile ground for future research. A large prospective cohort enrolling in Bangladesh, Tanzania, Uganda and Siberia may help to address this knowledge gap for some special population groups (NIH U01 ICIDR AI119954) but additional studies are needed.

Emerging and overdue priority areas for TB pharmacologists are specific design of antibiotic regimens for children and patients with extra-pulmonary TB. Frameworks are being developed for pediatric-focused HFS-based PK–PD studies [147] and it is to be hoped that clinical studies will follow.

Overall, despite numerous unanswered questions and technical challenges, the landscape has rarely been more optimistic in TB therapeutics. Judicious evaluation of novel antibiotic combinations carries real potential for better, safer and shorter treatment for all patient groups. Clinical PK–PD studies have an important role to play in achieving that goal.

**Executive summary**

**Background & rationale for clinical pharmacokinetic-pharmacodynamic studies of TB treatment**

- Shorter, less toxic treatment regimens are urgently required for all forms of TB.
- Clinical pharmacokinetic-pharmacodynamic (PK–PD) studies may help improve the conduct of Phase II clinical trials, and facilitate therapeutic drug monitoring for some drugs.

**Existing studies & their limitations**

- Results are variable but some show associations between low plasma C_Max_ or AUC measurement for first-line drugs and treatment response.
- Inconsistency in study design and selection of outcome measures contributes to difficulty in collective interpretation of prior data.

**Ongoing PK–PD work in drug-susceptible TB**

- Increased rifampicin doses for patients with pulmonary DS-TB may be associated with super-proportional increases in the AUC_0–24h_ and faster sputum sterilization.
- PK–PD modeling suggests that higher dose pyrazinamide may be possible.
- Fluoroquinolones doses may require to be increased to account for the effect of drug–drug interactions.

**Ongoing PK–PD work in multidrug resistant TB**

- The optimal dose of linezolid, to maximize efficacy while minimizing toxicity, is under evaluation.
- Therapeutic drug monitoring for aminoglycosides may be better targeted against C_Max_/MIC and AUC_0–24h_/MIC than traditional ‘peak’ and ‘trough’ measurements.
- Many additional questions require attention, including the efficacy and toxicity of novel drug combinations (e.g., bedaquiline and delamanid).

**Challenges for future studies**

- PK–PD study design should consider incorporating assays of active drug metabolites and free drug fractions. New treatment monitoring tools should be considered when selecting early outcome measures.
- PK–PD studies and clinical trials should be specifically designed for children and other special populations.
- PK–PD analyses at the site of infection may provide additional insights into the factors driving treatment efficacy.
